# Knee extensor muscle weakness and radiographic knee osteoarthritis progression: The influence of sex and malalignment

**DOI:** 10.1080/17453674.2018.1464314

**Published:** 2018-05-01

**Authors:** Andrea Dell’isola, Wolfgang Wirth, Martijn Steultjens, Felix Eckstein, Adam G Culvenor

**Affiliations:** a Institute of Applied Health Research/School of Health and Life Sciences, Glasgow Caledonian University, Glasgow, Scotland;; b Institute of Anatomy, Paracelsus Medical University Salzburg and Nuremburg, Salzburg, Austria;; c La Trobe Sport and Exercise Medicine Research Centre, School of Allied Health, La Trobe University, Bundoora, Australia

## Abstract

**Background and purpose — Knee extensor (KE) muscle weakness is a modifiable feature commonly observed in individuals with knee osteoarthritis (KOA) and constitutes a potential target for patient-specific interventions. Therefore, in this study, we explored whether KE weakness is associated with radiographic (medial and/or lateral) KOA progression and how this relationship differs depending on frontal plane knee alignment and sex.**

**Patients and methods — We studied 3,075 knees (1,961 participants, 58% female) from the Osteoarthritis Initiative with radiographic Kellgren–Lawrence grade 1–3. Peak KE torque (Nm/kg) was assessed at baseline, and progression defined as fixed-location joint space width loss (≥ 0.7mm) in medial and lateral tibiofemoral compartments from baseline to 4-year follow-up. Knee-based generalized estimating equations, stratified by alignment (malaligned vs. neutral), estimated the relative risk (RR) of progression for those in the lowest (and middle) vs. highest KE torque group (split by tertiles). Secondary analyses explored whether this relationship was compartmental- or sex-specific.**

**Results — Being in the lowest (or middle) compared with the highest torque group increased the risk of progression in neutrally aligned knees (relative risk [RR] 1.2 [95% CI 1.0–1.4]; and 1.2 [CI 1.0–1.4], respectively), but not after adjusting for age, sex, BMI, pain, and radiographic severity. In secondary analyses, women with neutral alignment in the lowest compared with the highest torque group had significantly increased risk of lateral compartment progression independent of age, BMI, disease severity, and pain (RR 1.3 [CI 1.0–1.8]). No association was observed between KE torque and KOA progression in men, irrespective of alignment.**

**Interpretation — These results identify a potentially important clinical phenotype: KE weakness may be a more important risk factor for radiographic KOA progression in women without knee malalignment.**

Knee extensor (KE) muscle weakness is a modifiable feature commonly observed in individuals with knee osteoarthritis (KOA) and a risk factor for incident radiographic KOA (Øiestad et al. 2015). In terms of disease progression, the relationship between KE weakness and radiographic KOA is less clear, perhaps due to an apparent sex-specific effect whereby women (but not men) with muscle weakness have an increased risk of KOA progression (Culvenor et al. 2017b).

Frontal plane knee alignment, an independent risk factor for KOA, has been reported to represent a potential confounder in the sex-specific relationship between KE weakness and KOA progression (Sharma et al. 2003). Contradictory to the concept of KE weakness increasing the risk of KOA progression, Sharma et al. (2003), in a study of 171 participants (328 knees), observed that greater KE torque increased the risk of radiographic KOA progression in individuals with established KOA and knee malalignment. These data provide preliminary evidence that the relationship between KE torque and disease progression depends on the local mechanical environment, where malalignment may determine how the medial and lateral tibiofemoral joint responds to muscle force. However, contradictory longitudinal MRI data in 265 older adults with KOA (mean age 67 years) subsequently showed that KE weakness did not influence medial and lateral compartment cartilage loss in either aligned or malaligned knees, but without accounting for previously observed differences in men and women (Amin et al. 2009).

Insights into the alignment- and sex-specific impact of KE weakness on medial and lateral KOA progression may be of value for developing personalized treatment strategies. Therefore, we determined whether the relationship between lower KE torque and risk of radiographic disease progression depends on knee alignment in a large cohort of >3,000 knees, and whether this relationship is compartment- and sex-specific. Based on previous data, we hypothesized that alignment modifies the relationship between KE torque and risk of radiographic progression and that women with KE weakness are at higher risk of KOA progression compared with men.

## Methods

### Participants

Participants were selected from the Osteoarthritis Initiative (OAI, http://www.oai.ucsf.edu), an ongoing multicenter cohort study in the United States designed to identify biomarkers and risk factors associated with KOA incidence and progression. The OAI includes 4,796 participants, aged 45–79 years, with, or at risk of, symptomatic KOA. For the current study, we included knees with radiographic Kellgren–Lawrence (KL) grade 1–3 at baseline (central reading release 0.7). Knees without any radiographic evidence of OA (KL 0) were excluded, as the current analysis focuses specifically on disease progression. Knees with end-stage disease (KL 4) were excluded due to having a limited capacity to progress. Eligible knees were those with KE strength, alignment (radiographic femur–tibia angle [FTA]) at baseline, and compartment-specific joint space width (JSW) measures recorded at baseline and 4-year follow-up (Neumann et al. 2009). From the entire 4,796 OAI participants (9,592 knees), a total of 3,178 participants (5,187 knees) had KL grade 1–3. Of these, 3,075 knees from 1,961 participants (58% female) were included in the current analysis (Table 1) (Additional File 2).

**Table 1.** Baseline characteristics of the sample ^a^

	All n = 1,961	Women n = 1,131	Men n = 830
Knees ^b^	3,075 (100)	1,825 (59.3)	1,250 (40.7)
Age, years	62.2 (8.8)	62.4 (8.7)	61.9 (9)
BMI, kg/m^2^	29.4 (4.7)	29.5 (5.1)	29.4 (4)
Height, m	1.68 (0.09)	1.63 (0.06)	1.76 (0.06)
Weight, kg	83.7 (16)	78.0 (14.5)	92.0 (14.3)
WOMAC, 0–100			
pain	12.9 (16.9)	14.2 (17.9)	11.0 (15.0)
physical function	13.1 (16.0)	16.0 (17.1)	10.6 (13.9)
stiffness	20.4 (20.3)	22.1 (21.1)	17.7 (18.7)
KL grade ^b^			
1	616 (20)	367 (20)	249 (20)
2	1,650 (54)	1,029 (56)	621 (50)
3	809 (26)	429 (24)	380 (30)
Knee extensor strength (N)	339 (129)	278 (93)	428 (24)
Knee extensor torque per			
body weight, Nm/kg	1.2 (0.4)	1.1 (0.4)	1.5 (0.5)
Frontal plane alignment, ᵒ	–1.1 (2.5)	–0.2 (2.3)	–2.5 (2.2)
Alignment ^b^			
Neutral	1,609 (52)	1,183 (65)	426 (34)
Varus	1,159 (38)	374 (20)	785 (63)
Valgus	307 (10)	268 (15)	39 (3)

BMI, body mass index; KL, Kellgren–Lawrence; WOMAC, Western Ontario and McMaster Universities Osteoarthritis Index.

**^a^**Except where indicated otherwise, values are mean (SD).

**^b^**Values are n (% of category)

## Evaluation of knee extensor muscle strength

Peak isometric KE strength was measured in N at baseline using the “Good Strength Chair” (Metitur Oy, Jyvaskyla, Finland) as described previously (Culvenor et al. 2016). Torque per body weight (Nm/kg) was calculated using the lever arm length recorded at the strength assessment. In the absence of previously defined thresholds, we used sex-specific tertiles of torque per body weight to create three equal-sized groups based on sex-specific KE torque (lowest, middle, highest group).

## Radiographic disease progression

Medial and lateral radiographic JSW was measured at baseline and follow-up with customized software at fixed locations, based on a range from X = 0% to X = 100% of the distal femur mediolateral width (central release 0.6) (Ornetti et al. 2009). For the current analysis, baseline fixed-location measures in the center of the medial (X = 22.5%) and lateral compartment (X = 80%) were used, as these were shown to display high sensitivity to change in KOA (Wirth et al. 2013).

Radiographic progression was defined as a JSW reduction of ≥0.7mm. This cut-off has been shown to provide the best predictive value for detecting progression at 3-year follow-up and was further validated using the Bland–Altman method to calculate the smallest detectable change (Bruyere et al. 2005, Ornetti et al. 2009). In these studies, a minimal JSW (mJSW) reduction of ≥0.7 mm in OAI control participants showed a minimal probability (≤ 10%) of change due to measurement error over a 12-month period (Ornetti et al. 2009). In the absence of a threshold for fixed location measures, we used the 0.7-mm threshold previously determined for mJSW; hence subjects with a reduction of JSW ≥0.7 mm in either the medial (X = 22.5%) or lateral (X = 80%) compartment were defined as progressors.

## Frontal plane knee alignment

Frontal plane knee alignment was assessed at baseline using the FTA, as previously described (release 0.6) (Iranpour-Boroujeni et al. 2014) (Additional File 1), which was more frequently recorded in the OAI database than the full-limb hip–knee–ankle (HKA) angle. More negative values indicate greater varus alignment. The FTA method has high intra- and inter-reader reproducibility (ICC ≥0.98) (Iranpour-Boroujeni et al. 2014) and is highly correlated with the HKA angle, having similar ability in predicting 2-year tibiofemoral cartilage loss (Iranpour-Boroujeni et al. 2014). For the current study, the HKA angle was derived from the FTA using sex-specific conversion formulae (Iranpour-Boroujeni et al. 2014). Alignment categories were valgus (HKA ≥ +2°), varus (HKA ≤ –2°), and neutral (HKA < ±2° from zero).

## Statistics

To adjust for correlations between limbs within each subject and to assess the risk of radiographic progression in subjects with lower KE torque, we used Poisson regression models with generalized estimating equations. An independent working correlation structure was used. To explore the effect of lowest (or middle) vs. highest KE torque (i.e., lowest vs. highest; middle vs. highest) on the risk of progression, we estimated the relative risk (RR) and 95% confidence intervals (CI) of KOA progression.

For the primary analysis, KOA progression was defined as occurring in either the medial or lateral tibiofemoral compartment and risk of progression was stratified by knee alignment (neutral vs. malaligned [i.e., either varus or valgus]) as per Sharma et al. (2003). Analyses were adjusted for confounders, selected using clinical reasoning and literature review, and portrayed in direct acyclic graphs to minimize over-adjustment and collider stratification bias. Analyses were adjusted for age, sex, BMI, KL grade, and baseline pain using knee-specific Western Ontario and McMaster Universities Osteoarthritis Index (WOMAC) pain score (0–100). Older age, sex, and knee-related pain increase risk of both KE weakness and KOA progression (Hunter 2009), while BMI and baseline KL grade directly influence KOA progression as well as being on a causal pathway via muscle weakness (i.e., BMI can influence KL grade which increases pain, leading to muscle weakness). We also adjusted for alignment (continuous variable to account for differences within each alignment category) as per Amin et al. (2009).

For secondary analyses, we calculated the compartment-specific risk of progression stratified by sex and knee alignment categories (neutral, varus, valgus). Knees without JSW reduction in either compartment were considered as non-progressors and used as controls. Analyses were adjusted as per primary analysis (excluding sex) and performed using SPSS (v22.0; IBM Corp, Armonk, NY, USA). p < 0.05 was considered statistically significant.

## Ethics, funding, and potential conflict of interest

Ethical approval and informed consent were obtained as part of the original OAI participant recruitment and data collection process. No specific ethical approval was therefore required for the current study.

The datasets analyzed during the current study are available in the Osteoarthritis Initiative (OAI) repository (https://oai.epi-ucsf.org/datarelease/).

The OAI is a public–private partnership comprising 5 contracts (N01-AR-2-2258; N01-AR-2-2259; N01-AR-2-2260; N01-AR-2-2261; N01-AR-2-2262) funded by the National Institutes of Health. Funding partners include Merck Research Laboratories; Novartis Pharmaceuticals Corporation; GlaxoSmithKline; and Pfizer, Inc. Private sector funding for the OAI is managed by the Foundation for the National Institutes of Health. This research has received funding from the European Union Seventh Framework Programme (FP7-PEOPLE-2013-ITN; KNEEMO) under grant agreement number 607510. AGC is a recipient of a National Health and Medical Research Council (NHMRC) of Australia Early Career Fellowship (Neil Hamilton Fairley Clinical Fellowship No. 1121173). The sponsors were not involved in the design and conduct of this particular study, in the analysis and interpretation of the data, and in the preparation, review, or approval of the manuscript.

Wolfgang Wirth has part-time employment with Chondrometrics GmbH and is a co-owner of Chondrometrics GmbH, a company providing MRI analysis services to academic researchers and to industry. Felix Eckstein is CEO of Chondrometrics GmbH; he has provided consulting services to Merck Serono, Samumed, and Bioclinica/Synarc, has prepared educational sessions for Medtronic, and has received research support from Pfizer, Eli Lilly, Merck Serono, Novartis, Stryker, Abbvie, Kolon, Synarc, Ampio, BICL, Orthotrophix, and Tissue Gene.

## Results

Women had 35% lower absolute muscle force (N) and 27% lower torque per body weight (Nm/kg) than men (Table 1). Approximately half of all knees had neutral alignment (52%), while 38% and 10% had varus and valgus malalignment, respectively (Table 1).

In the primary analysis, 1,431 knees (47%) displayed radiographic progression. 670 knees (22%) and 521 knees (17%) displayed medial and lateral progression, respectively, while 240 knees (7.8%) displayed progression in both compartments, and 1,644 (54%) no progression in either compartment. Being in the lowest or middle KE torque group increased the risk of KOA progression in either compartment for neutrally aligned knees in the unadjusted analysis (lowest KE torque group RR: 1.2 [1.0–1.4]; middle KE torque group RR: 1.2 [1.0–1.4]) but not after adjustment (Table 2). In malaligned knees, KE torque was not significantly associated with radiographic progression (Table 2).

**Table 2.** Risk of radiographic progression by sex-specific quadriceps torque group, stratified by knee alignment

Sex-specific torque group	Progressors n (%)	Crude RR (CI)	Adjusted ^a^ RR (CI)
Neutral alignment			
Low	226 (33)	1.16 (1.01–1.36)	1.03 (0.78–1.40)
Middle	241 (36)	1.18 (1.02–1.37)	1.12 (0.96–1.30)
High	209 (31)	1.00	1.00
Malalignment ^b^			
Low	253 (33)	1.13 (0.99–1.28)	1.00 (0.87–1.14)
Middle	256 (34)	1.05 (0.93–1.20)	0.99 (0.86–1.12)
High	246 (33)	1.00	1.00

RR: relative risk; CI: 95% confidence interval.

**^a^**Adjusted for baseline Kellgren–Lawrence grade, baseline WOMAC

pain, age, body mass index, sex, and alignment.

**^b^**Malalignment defined as ≥2° varus or valgus malalignment.

In secondary analyses, female knees without malalignment with the lowest KE torque had increased risk of radiographic progression in the lateral compartment compared with knees with the highest KE torque, both before and after adjustment (RRadj: 1.4 [1.0–1.8]). In contrast, KE torque did not significantly increase the risk of radiographic medial or lateral compartment progression in female knees with valgus or varus malalignment. Knee extensor torque also did not show any significant effect on the likelihood of KOA progression in men, regardless of the compartment and alignment (Table 3). However, due to the very low number of men with valgus malalignment (n = 39), it was not possible to perform analyses in male knees with valgus.

**Table 3.** Risk of radiographic progression by sex-specific extensor torque, stratified by sex and knee alignment

	Medial compartment			Lateral compartment		
Torque group	Progressors (%)**^a^**	Crude RR	Adjusted**^b^** RR	Progressors (%)**^a^**	Crude RR	Adjusted**^b^** RR
**Women**						
Neutral alignment						
Low	92 (33)	1.23 (0.93–1.62)	0.97 (0.71–1.32)	92 (37)	1.45 (1.08–1.94)	1.34 (1.04–1.84)
Middle	102 (36)	1.23 (0.94–1.60)	1.09 (0.83–1.44)	87 (34)	1.23 (0.92–1.65) 1.18 (0.87–1.60)	
High	86 (31)	1.00	1.00	73 (29)	1.00	1.00
Valgus alignment**^c^**						
Low	17 (32)	0.89 (0.46–1.72)	0.69 (0.33–1.44)	48 (37)	0.99 (0.73–1.35)	0.85 (0.61, 1.17)
Middle	21 (40)	1.12 (0.62–2.01)	0.99 (0.55–1.79)	43 (33)	0.90 (0.67–1.24)	0.87 (0.63–1.20)
High	15 (28)	1.00	1.00	38 (29)	1.00	1.00
Varus alignment**^c^**						
Low	50 (36)	1.31 (0.94–1.84)	1.18 (0.83–1.68)	20 (29)	1.10 (0.59–2.07)	0.92 (0.46–1.84)
Middle	44 (32)	1.18 (0.83–1.67)	1.18 (0.84–1.66)	28 (40)	1.57 (0.93–2.65)	1.45 (0.84–2.52)
High	44 (32)	1.00	1.00	21 (30)	1.00	1.00
**Men**						
Neutral alignment						
Low	44 (35)	1.02 (0.70–1.49)	0.97 (0.63–1.49)	48 (36)	1.11 (0.76–1.62)	1.07 (0.71–1.62)
Middle	42 (34)	1.08 (0.75–1.54)	1.08 (0.75–1.55)	45 (34)	1.15 (0.79–1.68)	1.12 (0.77–1.64)
High	39 (31)	1.00	1.00	39 (30)	1.00	1.00
Valgus alignment**^c^**						
Low	1 (12)	NA	NA	5 (33)	NA	NA
Middle	6 (76)	NA	NA	7 (46)	NA	NA
High	1 (12)	NA	NA	3 (20)	NA	NA
Varus alignment**^c^**						
Low	105 (34)	1.26 (0.96–1.58)	1.04 (0.82–1.32)	46 (28)	0.90 (0.62–1.31) 0.88 (0.60–1.30)	
Middle	100 (33)	1.06 (0.84–1.34)	0.92 (0.72–1.16)	56 (34)	0.97 (0.68–1.36) 0.94 (0.65–1.34)	
High	101 (33)	1.00	1.00	62 (38)	1.00	1.00

RR: relative risk; CI: 95% confidence interval.

**^a^**Percentage indicates progressors in the alignment category.

**^b^**Adjusted for baseline Kellgren–Lawrence grade, baseline WOMAC pain, age, body mass index, and alignment.

**^c^**Varus and valgus alignment defined as a variation ≥2° in either direction.

## Discussion

This is the first study to investigate the impact of KE weakness on the compartment- and sex-specific risk of radiographic KOA progression in the context of variation in frontal plane knee alignment. Our results from >3,000 knees suggest that lower KE torque is generally not associated with subsequent tibiofemoral radiographic progression, particularly when accounting for age, sex, BMI, radiographic severity, and pain. However, compartment- and sex-specific analyses revealed that lower KE torque was associated with lateral compartment progression in women with neutrally aligned knees (before and after adjustment). These results identify a potentially important clinical phenotype and highlight that KE muscle weakness may be an important risk factor for disease progression specifically in women without knee malalignment.

The findings of our primary analysis in neutral and malaligned knees do not confirm previous data from a smaller number of knees (n = 228) in the Mechanical Factors in Arthritis of the Knee (MAK) cohort that found KE weakness had a significant protective effect on KOA progression in malaligned knees (Sharma et al. 2003). In contrast, our results showed effect sizes of similar magnitude between neutral and malaligned knees, and that higher KE strength tended to be more (but not statistically significant) protective of KOA progression, as recently observed in a systematic review (Culvenor et al. 2017b). Our results extend this recent systematic review and meta-analysis that did not observe a significant association between KE weakness and tibiofemoral structural deterioration (Culvenor et al. 2017b) by accounting for variations in knee alignment and evaluating the sex-specific effect on medial and lateral compartment progression.

In stratifying by knee alignment categories in men and women, we observed that KE weakness increased the risk of (lateral) KOA progression in women with neutral knee alignment (before and after adjustment). That women, but not men, appear to be at increased risk of lateral tibiofemoral joint progression when KE weakness is present may be explained by women having a lower absolute strength capacity, and thereby potentially being closer to a threshold below which the risk of OA progression increases (Culvenor et al. 2017b). Moreover, KOA has a higher prevalence in women where biochemical differences (i.e., hormonal) are thought to play a role in the development and progression of disease, and the ability of KE muscle fibers to generate force diminishes with greater BMI in women, but not in men (Culvenor et al. 2017a).

Knee malalignment represents a well-established risk factor for KOA progression (Felson and Kim 2007), and, as hypothesized, the local mechanical environment appears to influence the relationship between KE torque and KOA progression demonstrated in previous work (Culvenor et al. 2016, Øiestad et al. 2015). It appears that once malalignment is present, the lack of KE torque has little effect on the risk of KOA progression. This is supported by the observation that 52% of all knees with malalignment displayed radiographic progression whereas only 42% did in the neutral group. In neutral aligned knees, in contrast, muscle weakness appears to play a role in subsequent progression, particularly in women, and therefore optimizing muscle impairments provides a potential avenue to help modify the risk of progression in women with neutral knee alignment.

Our findings somewhat contrast those of Amin et al. (2009) who observed no influence of sex-specific tertiles of KE torque on MRI-assessed medial or lateral cartilage lesion progression in knees without varus malalignment (i.e., < 5° varus). The inclusion of valgus knees in the “neutral aligned” group, the lack of stratification by sex, the different definition of progression (semi-quantitative MRI-based cartilage lesion scores), and the shorter observation period (15 to 30 months’ follow-up) may explain these differences and the lack of sensitivity in this previous study (Amin et al. 2009). Our findings of lateral compartment radiographic progression in female neutrally aligned knees may be the result of the differences in gait kinematics between sexes. Women with normal alignment have a larger knee abduction angle, hip adduction, and internal rotation during gait compared with men with normal alignment (Phinyomark et al. 2016). This specific kinematic pattern is thought to move the internal knee load towards the lateral compartment and has previously been associated with lateral KOA (Weidow et al. 2006). Women with muscle weakness therefore potentially have a higher risk of KOA progression in the lateral compartment due to the absence of muscle stability driving abnormal biomechanical load. Studies estimating knee internal contact forces are necessary in order to confirm this hypothesis.

Limitations of our study include the estimation of mechanical alignment from the FTA. However, a strong correlation between FTA and HKA suggests that similar results would occur irrespective of alignment assessment approach (Iranpour-Boroujeni et al. 2014). Despite the large study sample of OAI participants, we were unable to complete evaluations of the influence of KE weakness in men with valgus alignment due to the small number of men with knee valgus. The relatively small number of progressors in some other strata limits generalizability and means interpretation should be made with some caution. Second, to determine disease progression in both medial and lateral compartment we used the JSW threshold of 0.7 mm. This cut-off has been determined from test–retest measurements of medial compartment mJSW (Ornetti et al. 2009). However, since the mJSW can only be measured in the medial (and not the lateral) compartment, and because no cut-off has been established for fixed-location JSW measures, we decided to apply a cut-off of 0.7mm for fixed-location measures in the current study. Overall, generalizability of the results may be limited by cut-offs used to determine strata (i.e., muscle torque tertiles, alignment, and radiographic disease progression). In addition, considering the slow rate of progression that characterizes KOA, a longer follow-up period (> 4 years) should be considered to improve the estimates of the risk of OA radiographic progression associated with KE deficit. Finally, it is important to acknowledge that, in our secondary analysis, we did not account for multiple tests, but the analyses stratified by sex and compartment support the trends seen in the primary analyses.

Targeting the right patient with the right treatment constitutes a priority in KOA care (Dell’Isola et al. 2016). Optimizing muscle impairments could provide a potential avenue to help modify the risk of progression in women with neutral knee alignment. Exercise therapy, including muscle strengthening and neuromuscular exercises, is the first-line treatment for patients with KOA (Fernandes et al. 2013, National Institute of Clinical Excellence 2014). International guidelines indicate that these interventions need to be specifically tailored to the individual (Nelson et al. 2014). Results of our study suggest that including exercises aimed to increase KE torque may be particularly beneficial for structural outcomes in females with neutral alignment, in addition to optimizing functional capacity and reducing symptoms in all patients with KOA (Lange et al. 2008).

In summary, in the tibiofemoral joint of men and women as a whole, lower KE torque is generally not associated with KOA progression over 4 years, particularly after adjustment for other risk factors. However, in unravelling this relationship further, this study has identified an important subset of women (without malalignment), in which KE weakness was associated with (lateral) tibiofemoral progression. No relationship between KE weakness and compartment-specific progression was observed in men. Optimizing muscle impairments may help modify the risk of progression in women with neutral knee alignment.

## Supplementary data

Additional Files 1 and 2 are available as supplementary data in the online version of this article, http://dx.doi.org/10.1080/17453674.2018.1464314

All persons designated as authors qualify for authorship. Each author participated in the work and made substantial contributions to the manuscript.

The OAI is a public–private partnership comprising five contracts (N01-AR-2-2258, N01-AR-2-2259, N01-AR-2-2260, N01-AR-2-2261, and N01-AR-2-2262) funded by the National Institutes of health, a branch of the Department of Health and Human Services, and conducted by the OAI Study Investigators. This article was prepared using an OAI public use dataset and does not necessarily reflect the opinions or views of the OAI investigators or the NIH. The authors would like to kindly acknowledge “KNEEMO—Prevention and personalized treatments in knee osteoarthritis: an Initial Training Network” for the training.

*Acta* thanks Eva W Broström and other anonymous reviewers for help with peer review of this study.

## Supplementary Material

IORT_A_1464314_SUPP.pdf
