# Supplementary material for: Knee extensor muscle weakness and radiographic knee osteoarthritis progression: The influence of sex and malalignment
Source: Acta Orthop. 2018 May 1;89(4):406–11. doi: 10.1080/17453674.2018.1464314 (PMC6066769; doi:10.1080/17453674.2018.1464314)
Supplement: IORT_A_1464314_SUPP.pdf [file IORT_A_1464314_SM2701.pdf]

Supplementary data

Additional File 1

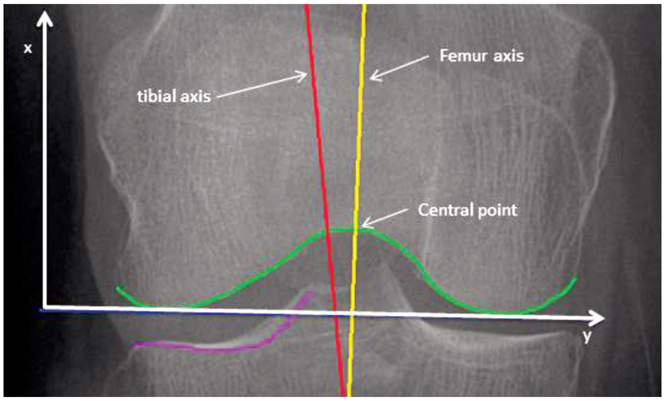

Femoral-Tibial Angle measurement description
The measurement of FTA involves the definition of the femoral axis using a coordinate system based on the shape of the femoral condyles that is defined as part of the location specific joint space width measurement. The femoral axis is defined to be perpendicular to the line tangent to the base of the femoral condyles. FTA is defined as the inclusion angle between the femoral axis and tibial axis, which was centred along the shaft of the tibia originating at a point 10 cm distal to the tibial plateau.

Additional File 2

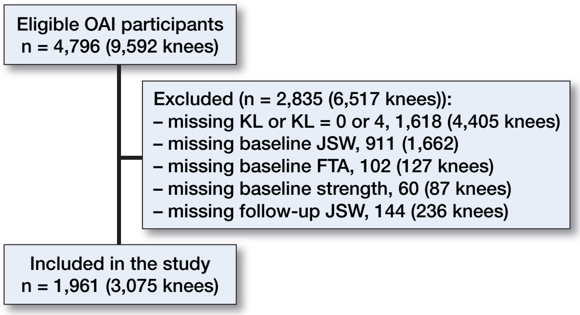

Participants selection flow chart
KL: Kellgren/Lawrence grade
JSW: joint space width
FTA: femoro-tibial angle
OAI: Osteoarthritis Initiative
